# Sexual attitudes and utilization of sexual and reproductive health services among older women in southern China: a qualitative study

**DOI:** 10.3389/fpubh.2024.1327734

**Published:** 2024-03-21

**Authors:** Xin Peng, Bingyi Wang, Qianyun Wang, Yuwei Li, Yinghui Sun, Xinyi Li, Joseph D. Tucker, Longtao He, Weiming Tang, Dan Wu, Huachun Zou

**Affiliations:** ^1^School of Public Health (Shenzhen), Sun Yat-sen University, Shenzhen, China; ^2^University of North Carolina Project-China, Guangzhou, China; ^3^Department of Social Welfare, University of California, Los Angeles, Los Angeles, CA, United States; ^4^Clinical Research Department, Faculty of Infectious and Tropical Diseases, London School of Hygiene & Tropical Medicine, London, United Kingdom; ^5^Research Institute of Social Development, Southwestern University of Finance and Economics, Chengdu, China; ^6^Dermatology Hospital of Southern Medical University, Guangzhou, China; ^7^Department of Social Medicine and Health Education, School of Public Health, Nanjing Medical University, Nanjing, China; ^8^School of Public Health, Fudan University, Shanghai, China; ^9^Shenzhen Campus, Sun Yat-sen University, Shenzhen, China; ^10^School of Public Health, Southwest Medical University, Luzhou, China; ^11^Kirby Institute, University of New South Wales, Sydney, NSW, Australia

**Keywords:** older women, sexual attitudes, sexual and reproductive health services, gender norms, China

## Abstract

**Objective:**

Sexual health plays a vital role in healthy aging. However, little is known about the sexual attitudes of and the utilization of sexual and reproductive health services by older women in China. This article is based on a qualitative study of older Chinese women in suburban areas to examine their attitudes toward sexuality and their utilization of sexual and reproductive health services.

**Methods:**

Face-to-face semi-structured interviews were conducted with older women (ages 50 to 74) from suburbs of southern China. Participants were purposively sampled on a convenience basis and recruited when they were visiting community health facilities between June and December 2021. Inclusion criteria were older women aged 50 years and older who had sexual experience. A topic guide was used that focused on sexual activity, sexual attitudes, the utilization of sexual and reproductive health services, and the factors that influence these. Interviews were audio recorded and transcribed verbatim. We coded the data inductively and conducted a thematic analysis.

**Results:**

Twenty-six Chinese women participated in the study. These older women had varying attitudes regarding sexual activity and its significance for older adults. The gender norms they held concerning sexual desire deemed that men had higher sexual desire than women. Most asymptomatic women did not actively seek sexual and reproductive health services. In most cases, women only sought professional services when they started to have sexual and reproductive health problems. Factors influencing the uptake of sexual and reproductive health services by older women were cost (affordability), availability, distance (accessibility), and conservative cultural norms towards sexuality.

**Conclusion:**

The attitudes of older women towards sexual activity are diverse. While some view sexual activity as common and essential for maintaining a sense of well-being in older age, others may hold different perspectives, considering it less significant. The utilization of sexual and reproductive health services by older Chinese women, except for when they were having a specific health issue, was low. Sexual health messages and services tailored for older women are needed.

## Introduction

Sexuality is a complex and multi-dimensional phenomenon, spanning biological, social, and physiological realms (1). As they age, some women become less interested in sexuality and focus more on caregiving (2, 3). However, others continue to have an active sexual life into older age and disapprove of the choice of a sexless lifestyle among older adults (4). The sexuality of older adults plays an important role in their lives (5), having an impact on their relationships with their partners and their quality of life. Sexual health is an integral part of the overall health of older people (6). The World Health Organization (WHO) defines *sexual health* as a state of physical, emotional, spiritual, and social well-being regarding sexuality; it is not just the absence of disease, dysfunction or infirmity. Supporting their sexual health arguably benefits the physical and mental health of older adults.

Previous studies have shown that older women may encounter more ageism than older men (7). Within cis-gender heterosexual couples, men often make critical decisions about sex, reducing opportunities for women to reflect on or have agency in their sexual lives (8). As a result, women are often excluded from discussions about sexuality and sexual health in clinical and other settings. However, contrary to popular assumptions, many older women are significantly sexually active and even engage in high-risk sexual behaviors (e.g., condomless sex) (9). Being sexually active in later life presents many challenges. For example, older adults constitute a growing number of those newly infected with sexually transmitted diseases (STDs) (10, 11), with syphilis diagnoses increasing significantly over time among older women. Because older women have a range of sexual health needs, it is important to focus on their sexual health and to lower the rates at which they are infected with STDs.

Although older adults are becoming more open-minded about their sexual attitudes, stereotypes regarding sexuality and old age persist (12). In traditional Chinese cultures, sexual and reproductive topics are not easily discussed and are sometimes regarded as taboo. In addition, it is difficult for Chinese women in rural areas to access free sexual and reproductive health services, especially after the age of 50 (13). Barriers to access, which are significant, can be both external (the system) and internal (personal). On the one hand, healthcare providers may hold prejudices that sex in later life is inappropriate or that older adults are asexual, and older adults may be aware of these prejudices (14–16). On the other hand, older women can be less likely to talk about sex and sexual health during medical visits (9).

There are very few studies about sex and sexual health attitudes among older women in China. Previous studies have shown that positive sexual attitudes can promote sexual health communication with healthcare providers (16) and encourage continued sexual activity among older women, which contributes to their overall physical and mental well-being (17, 18). In addition, sexual attitudes are sensitive variables in examining sexual function and are related to sexual health among middle-aged and older adults (19, 20). Given the growing recognition of the importance of sexuality to health and well-being, it is crucial to explore sexual attitudes among older women to promote their sexual health. To address the gap in the research, the purposes of our study were to (1) understand the sexual attitudes of and the utilization of sexual and reproductive health services by older women in southern China; (2) to illuminate the potential factors influencing different sexual attitudes and utilization of sexual and reproductive health services.

## Methods

### Study design

A qualitative study using in-depth semi-structured individual interviews was conducted between June and December 2021 among older women in two suburban areas (Baiyun District and Nansha District) of Guangzhou City, China, to gain a deeper understanding of the sexual attitudes in this population. The study was approved by the ethics committee of Sun Yat-sen University, and informed consent was obtained from the subjects.

### Data collection

Women aged 50 to 74 were purposively sampled on a convenience basis and recruited in person from two community health service centers. Inclusion criteria were Chinese women aged 50 or older who have ever had oral, vaginal, or anal sex. In our study, *sexually active* participants were defined as those who reported having engaged in sexual activity (oral, vaginal, or anal sex) in the past year.

Trained investigators introduced the program and invited older women who satisfied the eligibility requirements. Older women who consented to participate were interviewed face-to-face in a quiet and private space. Each interview lasted from 20 to 40 min. The interviews were anonymous and audio recorded after their consent had been obtained. Recordings were transcribed and the transcripts were checked by investigators for accuracy.

The topic guide was based on the relevant literature and developed following the guidance of experts from the local Center for Disease Control and Prevention. Basic demographic information and the general health status of the participants were collected in the interview. The topic guide focused on the following: sexual activity, sexual attitudes and the importance of sexual health, the utilization and factors influencing utilization of sexual and reproductive health services, and the participant’s understanding of sexual health and sources of information used. Given the sensitive nature of the topic of sex, we provided some incentives to relax the participants such as starting the interview with warm-up and non-sensitive questions (the general health status and mood of the participant).

### Analysis

We conducted a thematic analysis of the data following steps recommended by Braun and Clarke: becoming familiar with the data, coding data inductively, generating and reviewing themes, and defining and finalizing themes (21). We repeatedly read the preliminary transcripts, paying attention to the reflective and interpretive meanings therein and identifying the code categories list. Allowing these codes to evolve iteratively, preliminary themes were developed and modified as the data was analyzed. While focusing on recurring themes in the text, we also identified and highlighted cases that contradicted the dominant accounts to draw attention to differences in the experiences of older Chinese women. The key thematic categories were developed from summary statements, memos, and repeated reviews of the data. Data analysis was conducted using NVivo 11.0 (QSR International, Melbourne, Australia).

### Results

Interviews were conducted with 26 women (21 registered rural and 5 registered urban, median age = 66.5 years) (Table 1). Among them, 12 had a primary school education or below, and seven had a senior high school education or above. The majority were in stable relationships (married/cohabited with a partner, n = 15), and seven older women were in unstable relationships (widowed/currently separated/divorced). Nine older women were sexually active, and 17 were not. The following themes were identified: sexual attitudes, sexual experience, talking about sex, utilization of sexual and reproductive health services, and sources of sexual health information.

**Table 1:** Characteristics of interview participants.

Socio-demographic	Sample (*n* = 26)
Age, median (interquartile range)	66.5 (9.7)
Age group, years	
50–60	6
60–70	14
>70	6
Relationship status	
Stable relationship	15
Unstable relationship	11
Education level	
Primary school or below	12
Middle school	7
High school or above	7
Sexually active^*^	
No	17
Yes	9

^*^Sexually active participants were defined as those who reported sexual activity in the past year.

### Sexual attitudes

Older women had varying attitudes regarding sexual activity and its significance for older adults, including the degree to which sexual health contributed to healthy aging and whether or not it was normal for older people to engage in sex. Some sexually active older women believed that sexual behavior and sexual health were important among older adults, and they hoped that they could continue engaging in sexual activity for as long as their physical conditions permitted. These women explained that a good sexual experience can bring joy to their lives.

*I feel that older adults should pay attention to sexual and reproductive health because older adults also have sex. I think that there is no “older adults do not need (sex)” – this statement (NO.17, 54 years old, married, primary school or below education).*

*I think sexuality is very important for older adults. I now have a good sex life with my husband, about twice a week. I think sex makes our relationship harmonious and makes me happy, and I enjoy it. I also want to be able to keep having sex with my husband as long as our bodies allow (NO.13, 65 years old, married, high school or above education).*

Most single (widowed/divorced) older Chinese women in the study mentioned that older women typically lacked sexual desire or claimed that sexual activity, sexuality and sexual health were unimportant in the lives of older adults.

*I’m old, I have not had sex for a long time. Women do not want to have sex in their old age. What you said [about the importance of sexuality and sexual health] – I think is not necessary for my life (NO.6, 67 years old, married, primary school or below education).*

*My husband has been dead for years – I do not know what you mean by sexual health. We [older adults] do not learn about this stuff [sexual health] and do not understand it (NO.11, 74 years old, widowed, primary school or below education).*

Employment and education were considered to be factors influencing sexual health attitudes. In addition, regardless of whether they were sexually active, participants who had chronic medical conditions such as hypertension and diabetes prioritized their physical health over their sexual health.

*People who are employed, people who are educated, they pay attention to this [sexual health]. But those who come from the countryside to take care of their grandchildren, they do not understand [sexual health]. But I often want to help them or influence them. Where there is a physical examination, I will take them (NO.21, 64 years old, married, high school or above education)*.

*I think physical health is the most important thing for old people. I must take pills [treatment for chronic diseases] every day, which is a hassle. We do not care so much about sexual health. Sexual health for us older adults is not important (NO.24, 60 years old, married, middle school education).*

Gender norms concerning sexual desire, as perceived by older women, held that men had higher sexual desire than women. Only one older woman thought that men were generally under strain, working hard and having little free time and that this explained why their sexual desire was lower than that of women.

### Sexual experience

According to the women in our study, men made most of the decisions about cis-gender heterosexual sex rather than women, and women played a passive role. The majority of the sexually active older Chinese women in the study (*n* = 9) indicated that men initiated sex; only one participant reported that sexual activity happens spontaneously and that it did not require a certain party to take the initiative.

*My husband always actively performs sexual behavior. If he takes the initiative, I will satisfy him, and I rarely take the initiative (NO.22, 63 years old, married, high school or above education).*

*I still obey him [in terms of our sex life], sometimes two to three times a week. If he wants sex too many times, I will not [be willing]. I told him that someone said that when he reached 60 years old, he would not be able to have much of a sex life and that too much sexual behavior would make the short-lived. But I tell you that now is different from before – older adults who are generally seventy or eighty years old [still have sexual desire]. He [my husband] is now in his 60s, and his sexual desire is still very strong (NO.21, 64 years old, married, high school or above education).*

Most older women reported that they generally felt sexually satisfied and had positive sexual experiences. One participant mentioned she always had orgasms in her sexual encounters and enjoyed the sexual experience. She also believed that a good sex life made her happy and strengthened her relationship with her husband.

*The quality of sex is fine, and the frequency is just right, like 7 or 8 orgasms out of 10 times. We have a good relationship as a couple – I would not have sex with him if I did not like him, and I think he is very happy when he makes me orgasm. (NO.23, 50 years old, married, middle school education).*

Some older women said they had better sexual experiences when they were younger, but that aging, vaginal dryness, and decreased sexual desire have diminished these experiences. Many of these women with lower sexual desire or minor sex problems had sex only to satisfy the sexual needs of their partners.

*Vaginal dryness, I sometimes have this situation. When you are old, you may feel not as comfortable [having sex] as you did when you were young. I do not dislike sex. I will be satisfied when he needs it, but sometimes I am uncomfortable (NO.22, 63 years old, married, high school or above education)*

### Talking about sex

Whether or not they were sexually active, the majority of older women indicated that they never or hardly talked about sex or sexual health with their husbands or close friends. They even avoided listening to talk on the topic of sexuality because they felt it was too personal and uncomfortable to discuss. Interestingly, however, they claimed that it was acceptable to talk about sex privately with doctors or researchers (interviewers).

*There’s nothing to talk about [concerning sexual health]. We’re all healthy. My husband is very traditional. We do not talk about that (NO.25, 55 years old, married, primary school or below education).*

*I would not talk to my husband and friends about these topics [sexual health]. If I hear somebody talking about sex, I will leave. I think it is too embarrassing to talk about, and it is a very personal thing. But I think talking to you [about sex] is OK because you are a doctor [medical student] (NO.19, 65 years old, married, primary school or below education).*

A few sexually active older women claimed that they would talk about sex with their spouses or close friends to deepen their mutual understanding of their sex life and relationship, and said that they believed it was common for older adults to talk about sex.

*I would talk about it with my friends and ask about their sex life. I think it’s appropriate to talk about sex, and older adults should have a little bit of a sex life, right? (NO.17, 54 years old, married, primary school or below education).*

### Utilization of sexual and reproductive health services

Most asymptomatic women did not actively seek sexual and reproductive health services. In most cases, participants only attended gynecological examinations or sought professional services when they started to have sexual and reproductive health problems (e.g., mammary gland hyperplasia, uterine fibroids, and vaginal inflammation). The absence of reproductive system diseases and STDs was considered a crucial part of sexual health for some older women.

*I think the most important thing about sexual health is that there is no [reproductive] disease, no need to go to the doctor, and no need to take medicine. If I have a problem [disease], I will go to seek help [sexual/reproduction health services]. No matter how far it is, I will go to see them [doctors] (NO.6, 67 years old, married, primary school or below education).*

*Usually, I do not pay attention to these services [sexual and reproductive health services]. But I have uterine fibroids now, so I must be more concerned about gynecological health and need these services (NO.20, 58 years old, married, middle school education).*

Factors influencing participants’ utilization of sexual and reproductive health services were cost (affordability), availability, distance (accessibility), and conservative cultural norms towards sexuality. If the cost of sexual and reproductive health services is too high or if the older women are poor, they will not use such services.

*The cost [of using sexual and reproductive health services] is something I need to consider because I cannot use these services without money (NO.14, 67 years old, married, middle school education).*

Older women who want to and can afford the use of sexual and reproductive health services might consider the accessibility of the service location and might stop seeking such services if it were too far away or inaccessible. Some older women mentioned being influenced by traditional ideas; having relatively conservative values, they felt embarrassed to ask a doctor for sexual health advice and information.

*I know someone who would not go to the gynecology department, and then she said that she was afraid of being laughed at by others. At this age, you know, we are relatively conservative. She feels that talking about this topic [sexuality] is embarrassing, and sometimes she does not see a doctor, and she gets sick as a result (NO.26, 58 years old, married, high school or above education)*

### Sources of sexual health information

Qualitative results showed that sources participants used for information about sexual health included professionals, social media, relatives/friends, and official medical websites. Sometimes older women did not care or were not sure whether the information on the Internet was true or false – they just wanted to know a little about sexual health. Village committees also regularly informed older women of gynecological physical examinations in printed publicity notices and on social media (WeChat groups).

*I will search online [for sexual and reproductive health information]. In the doctor’s physical examination, he will teach you how to do a self-examination. I do not know whether the information on the Internet is accurate. Anyway, I just want to know how breast cancer occurs, to understand a little” (NO.22, 63 years old, married, high school or above education).*

## Discussion

The findings indicated that older women had varying attitudes regarding sexual activity and its significance to older adults. Older women who were sexually active and satisfied more likely to have positive and affirming sexual attitudes, recognize and value the importance of sexual activity and desire to continue sexual activity. In contrast, older women who were sexually inactive tended to have reserved or disinterested sexual attitudes, lack of sexual desire and perceive unimportance in sexual activity. Most older women had low utilization of sexual and reproductive health services and they tended to adopt a passive approach, only accessing professional services when specific issues or problems arose.

According to our findings, employment and education were influential factors influencing attitudes towards sex, consistent with previous studies that variables including social and economic status drive variations in the opinions and attitudes individuals hold toward sexuality (8). Possible explanations were that individuals with higher levels of education often have access to more comprehensive sexual education, and employment might provide opportunities for education and discussion about sexual health and wellness. Our results also demonstrated that high costs of services, and long distances (lack of transportation) to the health facility are barriers to accessing sexual and reproductive health services, which was similar to previous studies (22). These influencing factors need to be actively addressed to improve the utilization of sexual and reproductive health services. Moreover, older women with chronic diseases were more concerned with managing their physical pain or keeping track of their disease status than they were about looking after their sexual health. This resonates with findings from previous studies which indicate that poor physical condition, chronic disease, and related medications may diminish sexual performance and desire (23). In general, women who report having poor health are often less likely to engage in sexual activity, and those who do have sex are prone to report experiencing sexual difficulties (24). Therefore, actively enhancing the general health status of older women may contribute to improving their sexual attitudes and increase sexual engagement.

Among the older Chinese adults in our study, heterosexual sex was more often initiated by men than women, many of whom had little or no desire. Many of the older Chinese women reported that they only had sex to satisfy their partners. International studies are consistent with our findings. For example, according to results from qualitative interviews with 22 older adults from the third British National Survey of Sexual Attitudes and Lifestyles (Natsal-3), the most common sexual difficulty among older women is low or no sexual desire (25). An exploratory study of 454 married older individuals in Greece found that males are more likely to experience sexual desire and exhibit sexual behaviors (26). An existing systematic review of qualitative studies has reported that women are often subordinate and passive in sexual relations with men (8), especially in more conventional countries and societies in which the (hetero) sexual satisfaction of men is often considered to be the women’s obligation, and women play a passive role in sexual behavior (27–29).

This study indicated that many older women in China lacked essential sexual health information. This is supported by a survey conducted of 1,686 older rural Chinese women between the ages of 50 and 64. This survey revealed that, even though 54.4% of these women expressed the desire to increase their knowledge about sex and sexual health, only 9.3% of the health education these women had received in the previous 2 years was focused on advice about sex and contraception (13). The findings of our study demonstrated that not all older Chinese women were reserved and uninformed about sexual health; many of them expressed the desire to learn more about this subject. However, there are few resources for older adults to obtain this information, especially for older rural women (13). Appropriate lectures and publicity should be made more widely available, both online and offline. Sexual health information for older Chinese women should be tailored to their needs and delivered by health professionals and ensure that it is scientific and authentic. Such information should be designed to inform older adults that it is normal to have a sex life and to emphasize the importance of their sexual health.

When we interviewed older Chinese women about sex, we found that they were willing to talk to us about this otherwise unmentionable subject, in part because we were seen as “doctors,” after we had broken the ice by chatting informally. Most participants said that they avoided listening to other people discuss sexual topics and only occasionally or never talked about sex with their spouses or close friends. Previous studies have shown that when people have sexual health issues, communication about sex can help them renegotiate their sex lives and reduce feelings of distress (30, 31). Talking to spouses or friends about sensitive health-and relationship-related topics can also be a source of support for sexuality. In addition, as the first point of contact for older adults with sexual issues, healthcare providers play a major role in helping older adults enhance their sexual health (32). However, growing up in a culture in which sex was not openly discussed has been found to have harmed the sexual communication of older Australian adults, including their level of comfort in talking about sex (33). For older Chinese women, who might more conservative and traditional than older men, just talking about sex is taboo. Our study also found that conservative cultural norms may contribute to a sense of shame or embarrassment, preventing older women from openly discussing their sexual concerns with healthcare professionals or utilizing sexual and reproductive health services. It has been noted that healthcare providers can also be reluctant to bring up sexual health topics with patients for many reasons, including lack of time and knowledge, and the perception that the topic is inappropriate (34–36). Participants tended to use sexual and reproductive health services for gynecological examinations only. Such examinations could serve as an important entry point from which patients and doctors could engage in discussions about sexual health.

Our study has several limitations. First, our study design was based on a convenience sample of older women in suburban areas of southern China and all of the women were heterosexual. As such, it is uncertain how representative the attitudes of the participants towards sex are of the entire community or older women with other sexual orientations. Generalizations of results to other settings or populations may be limited. There is certainly scope for further investigation. Second, our study used thematic analysis, which may involve researchers’ subjective interpretation of the data. Strategies (37) have been adopted during data collection, analysis, and interpretation, including specialist training of data collectors before data collection; use survey topic guide to ensure consistency and clearly define research questions and objectives to guide data collection; multiple researchers in the coding process independently (XP and BW) and assess inter-coder reliability (QW and YL); share and discuss findings and interpretations with colleagues or experts (LH, WT and DW) to offer valuable insights. Third, our findings also need to be interpreted cautiously as information was collected based on self-reports and did not link participants’ views to their medical records, and information about the sexual health status and attitudes towards sex of the participants’ partners was not collect, which may have influenced the attitudes of the participants towards sex. Despite these limitations, the subjects of this study had different socio-demographic characteristics, ensuring diverse participant perspectives. Data collectors were well trained and follow standardized data collection protocols, which reduced variability. The researchers not only maintained reflexivity, acknowledging their preconceptions and biases, but also engaged in inter-coder reliability checks to ensure consistency in data interpretation, the investigator triangulation enhanced the credibility of the results (38). The consistency of our findings with existing studies on sexual health among older adults suggested transferability and confirmability. In addition, this qualitative study has given us insights into the sexual experiences and health of older Chinese women and was able to be used as a guide for a subsequent large-sample quantitative study: based on our study, our team conducted a multi-center epidemiological survey on the sexual health of older adults in China (39), to better understand the sexual health of community-dwelling older adults.

## Conclusion

In summary, the attitudes and experiences of older women towards sexual activity are diverse. While some view sexual activity as common and essential for maintaining a sense of well-being in older age, others may hold different perspectives, considering it less significant. The utilization of sexual and reproductive health services by older Chinese women, except for when they were having a specific health issue, was low. To improve their knowledge of sexual health and to enable them to achieve their desired sexual health status, it is essential to reduce the costs and enhance the accessibility, availability, and relevance of sexual health education for older adults and promote their use of sexual and reproductive health services.

## Data availability statement

The original contributions presented in the study are included in the article/supplementary materials, further inquiries can be directed to the corresponding authors.

## Ethics statement

The studies involving humans were approved by the study was approved by the ethics review board, School of Public Health (Shenzhen), Sun Yat-sen University (SYSU-PHS [2019]006) in China. The studies were conducted in accordance with the local legislation and institutional requirements. The participants provided their written informed consent to participate in this study.

## Author contributions

XP: Data curation, Formal analysis, Methodology, Writing – original draft. BW: Data curation, Investigation, Methodology, Writing – review & editing. QW: Data curation, Investigation, Methodology, Writing – review & editing. YL: Methodology, Writing – review & editing. YS: Methodology, Writing – review & editing. XL: Methodology, Writing – review & editing. JT: Data curation, Methodology, Writing – review & editing. LH: Data curation, Investigation, Methodology, Validation, Writing – review & editing. WT: Investigation, Validation, Writing – review & editing. DW: Conceptualization, Data curation, Software, Supervision, Validation, Writing – review & editing. HZ: Conceptualization, Funding acquisition, Resources, Supervision, Validation, Writing – review & editing.

## References

[1] Simpson P Horne M Brown LJ Wilson CB Dickinson T Torkington K. Old (Er) care home residents and sexual/intimate citizenship. Ageing Soc. (2017) 37:243–65. doi: 10.1017/S0144686x15001105, PMID: 28163343 PMC5244445

[2] Binfa L Robertson E Ransjo-Arvidson AB. Chilean Women's reflections about womanhood and sexuality during midlife in a Swedish or Chilean context. Health Care Women Int. (2009) 30:1093–110. doi: 10.1080/07399330903276777 19894153

[3] Kadri N Alami KM Berrada S. Sexuality in Morocco: women Sexologist's point of view. Theol Sex. (2009) 19:20–3. doi: 10.1016/j.sexol.2009.03.005

[4] Hinchliff S Gott M. Challenging social myths and stereotypes of women and aging: heterosexual women talk about sex. J Women Aging. (2008) 20:65–81. doi: 10.1300/J074v20n01_06, PMID: 18581701

[5] Ho PJ Goh YS. Health care professionals and care staff challenges and experiences of managing sexual expression among older adults >/=60 years in long-term care facilities: a qualitative review and meta-synthesis. Age Ageing. (2022) 51:afab230. doi: 10.1093/Ageing/Afab230, PMID: 34850812

[6] Ezhova I Savidge L Bonnett C Cassidy J Okwuokei A Dickinson T. Barriers to older adults seeking sexual health advice and treatment: a scoping review. Int J Nurs Stud. (2020) 107:103566. doi: 10.1016/J.Ijnurstu.2020.103566, PMID: 32380261

[7] Raqota B. Aging in America: ageism and general attitudes toward growing old and the elderly. Open J Soc Sci. (2017) 5:183. doi: 10.4236/jss.2017.58015

[8] Sinkovic M Towler L. Sexual aging: a systematic review of qualitative research on the sexuality and sexual health of older adults. Qual Health Res. (2019) 29:1239–54. doi: 10.1177/1049732318819834 30584788

[9] Thames A Hammond A Ra N Mahmood Z Jones F Sl C. Sexual health behavior and mental health among older African American women: the Sistahs, sexuality, and mental health well-being project. J Womens Health (Larchmt). (2018) 27:1177–85. doi: 10.1089/Jwh.2017.6777, PMID: 30070959

[10] Wang C Zhao P Xiong M Tucker JD Ong JJ Hall BJ. New syphilis cases in older adults, 2004-2019: an analysis of surveillance data from South China. Front Med. (2021) 8:781759. doi: 10.3389/Fmed.2021.781759, PMID: 34926524 PMC8674684

[11] Minichiello V Rahman S Hawkes G Pitts M. Sti epidemiology in the global older population: emerging challenges. Perspect Public Health. (2012) 132:178–81. doi: 10.1177/1757913912445688, PMID: 22729008

[12] Syme M. The evolving concept of older adult sexual behavior and its benefits. Generations. (2014) 38:35–41.

[13] Sun X Shu X Zong Z Mao J Sun Y Hearst N. Unmet sexual and reproductive health needs of women aged 50 to 64 years in rural China. Menopause. (2015) 22:505–11. doi: 10.1097/Gme.0000000000000346, PMID: 25349959

[14] Bauer M Haesler E Fetherstonhaugh D. Let's talk about sex: older People's views on the recognition of sexuality and sexual health in the health-care setting. Health Expect. (2016) 19:1237–50. doi: 10.1111/Hex.12418, PMID: 26448550 PMC6456814

[15] Fileborn B Lyons A Heywood W Hinchliff S Malta S Dow B. Talking to healthcare providers about sex in later life: findings from a qualitative study with older Australian men and women. Australas J Ageing. (2017) 36:E50–6. doi: 10.1111/Ajag.12450, PMID: 28639430

[16] Hughes AK Lewinson TD. Facilitating communication about sexual health between aging women and their health care providers. Qual Health Res. (2015) 25:540–50. doi: 10.1177/1049732314551062, PMID: 25228151

[17] Yee LA Sundquist KJ. Older Women's sexuality. Med J Aust. (2003) 178:640–3. doi: 10.5694/J.1326-5377.2003.Tb05393.X 12797854

[18] Inelmen EM Sergi G Girardi A Coin A Toffanello ED Cardin F. The importance of sexual health in the elderly: breaking down barriers and taboos. Aging Clin Exp Res. (2012) 24:31–4. 23160504

[19] Sierra JC Gómez-Carranza J Álvarez-Muelas A Cervilla O. Association of sexual attitudes with sexual function: general vs. specific attitudes. Int J Environ Res Public Health. (2021) 18:10390. doi: 10.3390/Ijerph181910390 34639691 PMC8508376

[20] Graf AS Patrick JH. The influence of sexual attitudes on mid-to late-life sexual well-being: age, not gender, As a salient factor. Int J Aging Hum Dev. (2014) 79:55–79. doi: 10.2190/AG.79.1.c 25486719

[21] Virginia B Victoria C. Using thematic analysis in psychology. Qual Res Psychol. (2006) 3:77–101. doi: 10.1191/1478088706qp063oa

[22] Nyalela M Dlungwane T. Men’ S utilisation of sexual and reproductive health services in low-and middle-income countries: a narrative review. South Afr J Infect Dis. (2023) 38:473. doi: 10.4102/Sajid.V38i1.473, PMID: 37435118 PMC10331170

[23] Merghati-Khoei E Pirak A Yazdkhasti M Rezasoltani P. Sexuality and elderly with chronic diseases: a review of the existing literature. J Res Med Sci. (2016) 21:136. doi: 10.4103/1735-1995.196618, PMID: 28331522 PMC5348839

[24] Kuhle CL Zhang X Kapoor E. Misconceptions about sexual health in older women: why we need to talk about it. Mayo Clin Proc. (2021) 96:866–9. doi: 10.1016/J.Mayocp.2020.09.037 33714605

[25] Hinchliff S Lewis R Wellings K Datta J Mitchell K. Pathways to help-seeking for sexual difficulties in older adults: qualitative findings from the third National Survey of Sexual Attitudes and Lifestyles (Natsal-3). Age Ageing. (2021) 50:546–53. doi: 10.1093/Ageing/Afaa281, PMID: 33507242 PMC7936020

[26] Papaharitou S Nakopoulou E Kirana P Giaglis G Moraitou M Hatzichristou D. Factors associated with sexuality in later life: an exploratory study in a group of Greek married older adults. Arch Gerontol Geriatr. (2008) 46:191–201. doi: 10.1016/J.Archger.2007.03.008, PMID: 17532065

[27] Yun O Kim M SE C. The sexuality experience of older widows in Korea. Qual Health Res. (2014) 24:474–83. doi: 10.1177/1049732313518978, PMID: 24406484

[28] Baldissera VDA Bueno SMV Hoga LAK. Improvement of older Women's sexuality through emancipatory education. Health Care Women Int. (2012) 33:956–72. doi: 10.1080/07399332.2012.684986, PMID: 22946596

[29] Lagana L Maciel M. Sexual desire among Mexican-American older women: a qualitative study. Cult Health Sex. (2010) 12:705–19. doi: 10.1080/13691058.2010.482673, PMID: 20526982 PMC2997623

[30] Frederick DA Lever J Gillespie BJ Garcia JR. What keeps passion alive? Sexual satisfaction is associated with sexual communication, mood setting, sexual variety, Oral sex, orgasm, and sex frequency in a National U.S Study. J Sex Res. (2017) 54:186–201. doi: 10.1080/00224499.2015.1137854 26900897

[31] Gillespie BJ. Correlates of sex frequency and sexual satisfaction among partnered older adults. J Sex Marital Ther. (2017) 43:403–23. doi: 10.1080/0092623x.2016.1176608, PMID: 27115100

[32] Hughes AK Rostant OS Curran PG. Improving sexual health communication between older women and their providers: how the integrative model of Behavioral prediction can help. Res Aging. (2014) 36:450–66. doi: 10.1177/0164027513500055, PMID: 25651316

[33] Hinchliff S Fileborn B Alba B Lyons A Minichiello V Barrett C. Talking about sex with friends: perspectives of older adults from the sex, age & me Study in Australia. Cult Health Sex. (2021) 23:367–82. doi: 10.1080/13691058.2019.1710568, PMID: 32609066

[34] Dyer K das Nair R. Why Don't healthcare professionals talk about sex? A systematic review of recent qualitative studies conducted in the United Kingdom. J Sex Med. (2013) 10:2658–70. doi: 10.1111/J.1743-6109.2012.02856.X, PMID: 22846467

[35] Mcgrath M Lynch E. Occupational Therapists' perspectives on addressing sexual concerns of older adults in the context of rehabilitation. Disabil Rehabil. (2014) 36:651–7. doi: 10.3109/09638288.2013.805823, PMID: 23802139

[36] Hinchliff S Gott M. Seeking medical help for sexual concerns in mid-and later life: a review of the literature. J Sex Res. (2011) 48:106–17. doi: 10.1080/00224499.2010.548610, PMID: 21409708

[37] Castleberry A Nolen A. Thematic analysis of qualitative research data: is it as easy as it sounds? Curr Pharm Teach Learn. (2018) 10:807–15. doi: 10.1016/J.Cptl.2018.03.019, PMID: 30025784

[38] Korstjens I Moser A. Series: practical guidance to qualitative research. Part 4: trustworthiness and publishing. Eur J Gen Pract. (2018) 24:120–4. doi: 10.1080/13814788.2017.1375092, PMID: 29202616 PMC8816392

[39] Wang B Peng X Liang B Fu L Tian T Liu J. Sexual well-being among older adults in China (swell): protocol for a Multicenter cross-sectional study. BMJ Open. (2023) 13:E67338. doi: 10.1136/Bmjopen-2022-067338, PMID: 36717139 PMC9887691

